# Effects of nigella sativa oil on allergic rhinitis: an experimental animal study

**DOI:** 10.1016/j.bjorl.2022.09.003

**Published:** 2022-09-21

**Authors:** Recep Gul, Hasan Deniz Tansuker, Abdurrahman Bugra Cengiz, Melda Gul, Alper Tabaru, Funda Emre, Mehmet Faruk Oktay

**Affiliations:** a University of Health Sciences Kanuni Training and Research Hospital, Department of Otorhinolaryngology, Istanbul, Turkey; b Yeditepe University, Faculty of Medicine, Department of Otorhinolaryngology, Istanbul, Turkey; c University of Health Sciences Bagcilar Training and Research Hospital, Department of Otorhinolaryngology, Istanbul, Turkey; d Mehmet Akif Cardiovascular and Thorasic Surgery Training and Research Hospital, Department of Otorhinolaryngology, Istanbul, Turkey; e Basaksehir Cam and Sakura State Hospital, Department of Otorhinolaryngology, Istanbul, Turkey; f University of Health Sciences Bagcilar Training and Research Hospital, Department of Pathology, Istanbul, Turkey

**Keywords:** Allergic rhinitis, Mometasone furoate, Nigella sativa oil

## Abstract

**Objectives:**

Nigella sativa oil is known antiallergic and immunomodulatory effects. We aimed to compare this oil with mometasone furoate, a topical steroid, on a rat model in the prevention of allergic rhinitis symptoms.

**Methods:**

A total of 28 two-to-four-month-old Wistar Hannover rats weighing 250–350 g were randomly divided into four groups of seven, which included control, allergic rhinitis, mometasone furoate, and Nigella sativa oil groups. Loss of cilia, an increase of goblet cells, vascular proliferation, inflammatory cell count, eosinophil infiltration, and the degree of hypertrophy in chondrocytes were assessed by light microscopy.

**Results:**

The frequency of nasal scratching in the Nigella sativa oil group was found to be significantly lower compared with the allergic rhinitis group (*p* < 0.05). Typical inflammatory changes seen in allergic rhinitis were not detected in the Nigella sativa oil group. No inflammation was observed in 85.7% of both the healthy control group and the Nigella sativa oil group. In addition, no inflammation was observed in 71.4% of the mometasone furoate group, and this difference was found to be significant compared with the control group (*p* < 0.05). In addition, eosinophil infiltration, cilia loss, chondrocyte hypertrophy, vascular proliferation, and goblet cell increase were found to be significantly decreased in the mometazone furoate and Nigella sativa oil groups compared to the allergic rhinitis group (*p* < 0.05).

**Conclusion:**

According to the findings obtained from this study, we found anti-inflammatory and anti-allergic effects of Nigella sativa oil as equally effective as mometasone furoate in the treatment of experimentaly generated allergic rhinitis.

**Level of evidence:**

IV.

## Highlights

• Nigella sativa oil has an anti-inflammatory efficacy on allergic rhinitis in animal model
• Nigella sativa oil has similar effects with Mometasone furoate on allergic rhinitis in animal model
• The frequency of sneezing and nose scratching movements decreased significantly in animals treated with Nigella sativa oil

## Introduction

Allergic Rhinitis (AR) is an inflammatory disease of the nasal mucosa mediated by immunoglobulin (Ig) E, which often causes symptoms such as nasal discharge, itching, sneezing, and congestion. It is estimated to affect approximately 20%–40% of the world’s population.^1^ AR negatively affects school, work activities, and social life by causing distraction, snoring, and sleep disorders, while also negatively impacting cognitive functions. In addition, it has become a large-scale public health problem due to the medical needs that emerge in the course of the disease and the economic burden it causes due to missed work/school days and productivity losses.^2^, ^3^, ^4^ Drugs used in AR treatment can be listed as topical and oral corticosteroids, topical and oral antihistamines, topical and oral decongestants, mast cell stabilizers, mucolytics, anticholinergic agents, and systemic immunotherapy, each of which can change the course of the disease.^5^

The increasing interest in natural products over time and the concerns about the side effects of traditional medicines have brought the use of herbal medicines to the forefront as an alternative in the treatment and cure of various diseases. One of the more increasingly popular medicinal plants in recent years has been Nigella sativa (N. sativa), which has a rich history. N. sativa seeds were used by ancient Egyptian and Greek doctors to treat headache, toothache, nasal congestion, and intestinal worms; to support menstruation; as a diuretic; and to increase milk production in the postpartum period. In Central and East Asia, N. sativa seeds have been used in the treatment of many diseases, such as bronchial asthma, headache, dysentery, infections, obesity, back pain, hypertension, and gastrointestinal problems.^6^

We aimed to compare the effectiveness of the topical use of mometasone furoate, which is a topical corticosteroid used in the first-line treatment of AR, and N. sativa oil, which is also known to be antiallergic and anti-inflammatory, in the prevention of disease symptoms in a rat AR model.

## Methods

This study was carried out in the Istanbul Bagcilar Training and Research Hospital Experimental Animal Research and Application Center. Ethics committee approval was received from the Experimental Animals Ethics Committee of the same center (nº 2018-45). A total of 28 Wistar Hannover rats weighing 250–350 g, two to four months old, were randomly divided into four groups of seven. All of the rats were male gender to avoid hormonal differences and possibilities of interference in the results.

Table 1 shows the grouping. Group 1 was the control group, and Groups 2 through 4 were AR groups. Group 2 was the AR group, to which no treatment was given, Group 3 was treated with mometasone furoate, and Group 4 was given N. sativa oil. All animals were kept at room temperature of 22 °C on a 12-h dark/light cycle. Animals in all groups were fed freely with standard feed, and fresh drinking water was given to all groups every day.

**Table 1:** Description and Number of animals in groups.

Experimental groups	Number of animals
Group 1 Control group	7
Group 2 Allergic rhinitis control group	7
Group 3 AR Group given mometasone furoate treatment	7
Group 4 AR Group given Nigella sativa oil	7

The drugs used in the study were weighed with a Sartorius brand precision balance (GD603-0CE Carat Scale, Sartorius Mechatronics, Goettingen, Germany). Animals in Groups 2, 3 and 4 were given 0.3 mg ovalbumin (OVA, Grade V, Sigma-Aldrich Chemical Co. St. Louis, MO) and 30 mg aluminum hydroxide as an adjuvant in 1.0 mL 0.9% Saline (SF) described previously.^7^ The prepared antigen solution was given intraperitoneally and applied seven times for a total of 14 days, once every two days to create sensitization. To the animals in the control group, 1.0 mL 0.9% SF was administered intraperitoneally seven times once every two days for a total of 14 days.

In the second phase of the study, to create an AR model in the sensitized animals, 1.0 mg/mL OVA in 0.9% SF and 0.54 U protease from Aspergillus oryzae was centrifuged, and 30 μL of the solution was instilled into both nostrils with a micropipette every day for 15 days.

In addition to the OVA + protease application, 10 μL of mometasone furoate solution prepared as 5.0 mcg/kg was given intranasally to the animals in the third group every day for 15 days, one hour before OVA application, with a micropipette. The animals in the fourth group were also administered the OVA + protease application but were fed with fodder that contained 2.0 mL/kg N. sativa oil. For the animals in the control group, 10 µL of 0.9% SF was instilled into both nostrils with a micropipette every day for 15 days. No animals died during the experiment.

Twenty-four hours after the drug administration, all animals were sacrificed by intraperitoneal pentobarbital injection of 80 mg/kg and perfused with 100 mL 0.9% SF and then 400 mL formaldehyde in the left ventricle. Then, the nasal cavity, nasal septum, paranasal sinus, and concha of the animals were removed as a block.

Subjective evaluation of AR symptoms was done on the 1st, 14th, 17th, 20th, 23rd, 26th, and 28th days. Immediately after the intranasal OVA application, one animal in each cage was subject to a 10-minute adaptation period, which was attended to by the same person for 10 min to observe the number of nose scratchings and sneezing. The animals were scored by evaluating the nose scratching movement and the number of sneezes on the 14th day, which is the day accepted as the onset of local sensitization. As shown in Table 2, the numbers of sneezing and nose scratching movements were counted separately and scored from 0 to 3 points. and a successful AR model was determined if the total score was >5 points described previously.^8^

**Table 2:** Scoring table for the number of nose scratches and sneezes.

	0 Points	1 Points	2 Points	3 Points
**Number of nose scratching movements**	Unobserved	2 times/min	4–6 times/min	>6 times/min
**Number of sneezes**	Unobserved	2 times/min	4–6 times/min	>6 times/min

**Figure 1:**
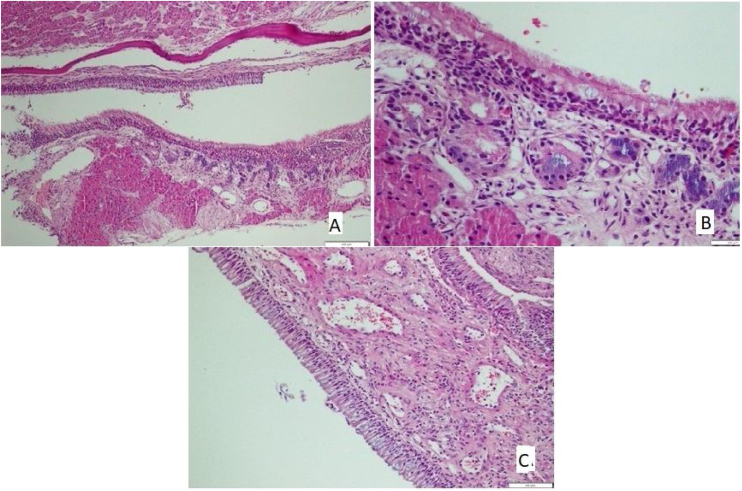
(A) Goblet cell hyperplasia, increased inflammation, loss of cilia, and increased eosinophils in Allergic Rhinit model (HE × 40). (B) Congestion and increased vascularisation in Allergic Rhinit model (HE × 40). (C) Chondrosithipertrophy in Allergic Rhinit model (HE × 40).

The tissues removed from nasal mucosa were dehydrated and embedded in paraffin, stained with hematoxylin and eosin after sectioning at 4 µm thickness, and analyzed with light microscopy. Loss of cilia, an increase of goblet cells (Fig. 1A), vascular congestion, vascular proliferation (Fig. 1B), inflammatory cell infiltration, eosinophil infiltration, and chondrocyte hypertrophy (Fig. 1C) were assessed by light microscopic examination; Scoring followed the protocol of Ercan et al.^9^ and was performed semi-quantitatively for each parameter by assigning “0” if there was no change, “1” if there was a slight change, “2” if there was a significant change, and “3” if there was a severe change.^10^

### Statistical analysis

Mean, standard deviation, and median values were used in the descriptive statistics of the data. The compliance of variables to normal distribution was measured with the Kolmogorov-Smirnov test. Crosstab (Chi-Square) analysis was used for the non-numerical variables. The Kruskal-Wallis H test, which is used among three or more groups that do not show a normal distribution, was implemented in the analysis of the numerical variables to determine the number of instances of sneezing and nose scratching. The Mann-Whitney *U* test was used to assess the groups with significant differences between them. The results obtained were evaluated at a 95% (*p* < 0.05) significance level.

## Results

### Subjective assessment of symptoms

It was observed that the number of sneezing and nose scratching incidents in rats did not follow the normal distribution on any day starting from 14th day (*p* = 0.05, *p* = 0.03, *p* = 0.02, respectively for 14th, 20th, and 28th days). In the evaluation of AR, it was accepted that a successful AR model had been provided in all animals in the second, third, and fourth groups since the total AR score was higher than five from the 14th day. Thus, all animals in these groups were included in the study.

The difference between the averages of the groups in terms of the number of sneezing instances on the 1st day was not statistically significant (*p* > 0.05). There was, however, a statistically significant difference between the averages of the groups in terms of the number of sneezes on days 14, 17, 20, 23, 26, and 28 (*p* < 0.05).

It was observed that the number of sneezes in the AR group on days 14, 17, 20, 23, 26, and 28 was statistically significantly higher than sneezes on the first day, while there was no significant change in the control group (*p* = 0.001, *p* = 0.002, *p* = 0.001, *p* = 0.001, *p* = 0.001, and *p* = 0.001, respectively). In comparison, the number of sneezes on days 14, 17, 20, 23, 26, and 28 in the mometasone furoate group was found to be significantly higher than those on the first day compared with the control group (*p* = 0.001, *p* = 0.000, *p* = 0.001, *p* = 0.001, *p* = 0.001, and *p* = 0.001, respectively).

The number of sneezes in the Nigella sativa oil group on days 14, 17, 20, 23, 26, and 28 combined was found to be significantly higher than those on the first day compared with the control group (primary *p* = 0.001, *p* = 0.000, *p* = 0.000, *p* = 0.001, *p* = 0.001, and *p* = 0.001). In the mometasone furoate group, the number of sneezes on days 17, 20, 23, 26, and 28 was statistically significantly lower than those on days 1 and 14 compared with the AR group (*p* = 0.007, *p* = 0.002, *p* = 0.002, *p* = 0.002, and *p* = 0.002, respectively).

It was observed that the number of sneezing instances on days 17, 20, 23, 26, and 28 was statistically significantly lower than those on the first and 14th days in the Nigella sativa oil group when compared with the AR group (*p* = 0.012, *p* = 0.001, *p* = 0.001, *p* = 0.001, and *p* = 0.001, respectively). In the comparison of the mometasone furoate and Nigella sativa oil groups, we found no significant difference in the number of sneezes between days (*p* = 0.122, *p* = 0.700, *p* = 1.000, *p* = 0.477, *p* = 0.790, *p* = 0.840, and *p* = 0.630, respectively).

Nose scratching frequency in the AR group on days 14, 17, 20, 23, 26, and 28 was found to be higher than the number of nose scratching instances on the first day (*p* = 0.018, *p* = 0.018, *p* = 0.018, *p* = 0.018, *p* = 0.018, and *p* = 0.018, respectively). In the mometasone furoate group, the nose scratching frequency of the rats on days 14, 17, 20, 23, 26, and 28 was significantly higher than on the first day (*p* = 0.018, *p* = 0.017, *p* = 0.025, *p* = 0.025, *p* = 0.020, and *p* = 0.014, respectively).

Nose scratching frequency on the 14th and 17th days was significantly higher than the number of nose scratches on the first day in the Nigella sativa oil group (*p* = 0.018 and *p* = 0.018, respectively). It was also found that nose scratching frequency on days 20, 23, 26, and 28 was not statistically different compared with the first day (*p* = 0.059, *p* = 0.317, *p* = 0.276, and *p* = 0.655, respectively).

Compared with the control group, nose scratching frequency in the AR group on days 14, 17, 20, 23, 26 and 28 was found to be statistically significantly higher than the rate on day 1 (*p* = 0.001, *p* = 0.001, *p* = 0.001, *p* = 0.002, *p* = 0.002, *p* = 0.001, respectively). Further, the number of sneezes in the mometasone furoate group on days 14, 17, 20, 23, 26 and 28 was found to be statistically significantly higher than the frequency on the first day (*p* = 0.001, *p* = 0.000, *p* = 0.001, *p* = 0.001, *p* = 0.001, and *p* = 0.001, respectively).

We observed that the number of sneezing instances on days 14, 17, 20, 23, 26 and 28 in the Nigella sativa oil group was statistically significantly higher than those on the first day compared with the control group (*p* = 0.001, *p* = 0.000, *p* = 0.000, *p* = 0.001, *p* = 0.001, and *p* = 0.001, respectively). Compared with the AR group, the number of sneezes on days 17, 20, 23, 26, and 28 was statistically significantly lower than those on the 1st and 14th days in the mometasone furoate group (*p* = 0.007, *p* = 0.002, *p* = 0.002, *p* = 0.002, and *p* = 0.002, respectively).

There was no statistical difference in the number of sneezing days in comparison to the mometasone furoate and Nigella sativa oil groups.

The comparison of the sum scores of sneezing and nose scratching frequencies and significant decrease of the symptoms after Nigella sativa oil and mometasone furoate of the groups on the 28th day was shown in Table 3.

**Table 3:** Mean nose scratching and sneezing scores of the groups on the 28th day.

	Groups			
	Control group	Allergic rhinitis group without treatment	Mometazone furoate given group	Nigella sativa oil given group
**Mean of the Nose Scratching and sneezing scores (standard deviation)**	1 ± 0.22	5.28 ± 1.1	1.71 ± 0.8	1.857 ± 0.23
**p-value for 28^th^ day**		0.001^a^	0.001^b^	0.001^c^

a Comparison of the experimental allergic rhinitis group with the the control group.

b Comparison of the mometasone furoate given group with the allergic rhinitis group.

c Comparison of Nigella sativa oil given group with the allergic rhinitis group.

### Histological evaluation

Mild inflammation was observed in 14.3% of the control group, AR group, and Nigella sativa oil group; however, 28.6% of the mometasone furoate group had mild inflammation levels. No inflammation was seen in 85.7% of the control group and Nigella sativa oil group and 71.4% of the mometasone furoate group. The difference between the groups was found to be significant (*p* < 0.05).

Eosinophil infiltration was found to vary between the groups, and this variance was statistically significant (*p* < 0.05; Table 4).

**Table 4:** Comparison of eosinophil infiltration by groups. Crosstab (Chi-Square) analysis was used for the non-numerical variables The results obtained were evaluated at a 95% (*p* < 0.05) significance level (n = number).

		Groups				Total	*p*-value*
		Control group	Allergic rhinitis group without treatment	Mometazone furoate given group	Nigella sativa oil given group		
**Eosinophil infiltration**							0.005
Unobserved	n	7	0	6	3	16	
	%	100.0	0.0	85.7	42.9	57.1	
Very rare	n	0	4	1	3	8	
	%	0.0	57.1	14.3	42.9	28.6	
Frequent	n	0	3	0	1	4	
	%	0.0	42.9	0.0	14.3	14.3	
**Total**	n	7	7	7	7	28	
	%	100.0	100.0	100.0	100.0	100.0	

**Figure 2:**
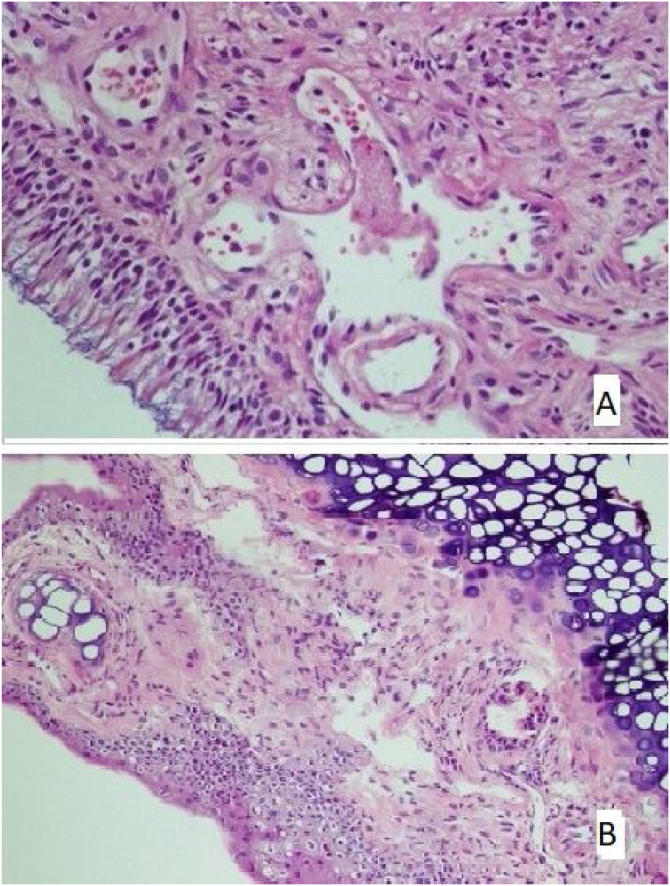
(A) The section in which the cilia are clearly observed, and there is no inflammation and eosinophil increase in the mometasone furoate treatment group(HE × 40) and (B) the section where inflammation is not observed, cilia are preserved, and goblet cell hyperplasia is not observed in the Nigella sativa oil treatment group (HE × 40).

There was no loss of cilia in all (100%) rats in the control and mometasone furoate groups and 28.6% of the rats in the Nigella sativa oil group. It was observed that 42.9% of those in the AR group had a mild loss of cilia, and 57.1% had a moderate loss, whereas 71.4% of those in the Nigella sativa oil group had a mild loss (Fig. 2A). This difference between the groups was statistically significant (*p* < 0.05)

The increase in goblet cells was seen to significantly vary between the groups (*p* < 0.05; Table 5). It was observed that 71.4% of the rats in the control group, 42.9% of the rats in the mometasone furoate group, and 14.3% of the rats in the Nigella sativa oil group did not have vascular proliferation. (Fig. 2B) Mild vascular proliferation was observed in 28.6% of the control and AR groups, 57.1% of those in the mometazonfuroate group, and 71.4% of those in the Nigella sativa oil group. In addition, it was observed that 71.4% of the AR group and 14.3% of the Nigella sativa oil group had moderate vascular proliferation. This difference between the groups was statistically significant (*p* < 0.05).

**Table 5:** Comparison of goblet cell increase by groups Crosstab (Chi-Square) analysis was used for the non-numerical variables The results obtained were evaluated at a 95% (*p* < 0.05) significance level (n = number).

		Groups				Total	*p*
		Control group	Allergic rhinitis group without treatment	Mometazone furoate given group	Nigella sativa oil given group		
**Goblet cell increase**							0.001
Unobserved	n	7	0	5	3	15	
	%	100.0	0.0	71.4	42.9	53.6	
Very rare	n	0	2	2	4	8	
	%	0.0	28.6	28.6	57.1	28.6	
Frequent	n	0	5	0	0	5	
	%	0.0	71.4	0.0	0.0	17.9	
**Total**	n	7	7	7	7	28	
	%	100	100	100	100	100	

Differences in hypertrophic chondrocyte levels were observed between the groups, and this result was statistically significant (*p* < 0.05; Table 6).

**Table 6:** Comparison of the hypertrophy levels in the chondrocytes according to the groups. Crosstab (chi-square) analysis was used for the non-numerical variables The results obtained were evaluated at a 95% (*p* < 0.05) significance level (n = number).

		Groups				Total	*p*
		Control group	Allergic rhinitis group without treatment	Mometazone furoate given group	Nigella sativa oil given group		
**Chondrocyte hypertrophy**							0.000
Unobserved	n	7	0	7	3	17	
	%	100.0	0.0	100.0	42.9	60.7	
Very rare	n	0	7	0	4	11	
	%	0.0	100.0	0.0	57.1	39.3	
Frequent	n	0	0	0	0	0	
	%	0.0	0.0	0.0	0.0	0.0	
**Total**	n	7	7	7	7	28	
	%	100.0	100.0	100.0	100.0	100.0	

## Discussion

AR is a general health problem due to its high prevalence and high comorbidity and complications.^11^ AR negatively affects a person’s quality of life, psychological well-being, cognitive functions, and sleep quality. It has been reported that patients frequently experience disturbing symptoms, mood disorders, decreased work productivity, and even job loss.^12^ Although there have been significant developments regarding AR concerning physiopathology and treatment, discussions about appropriate treatment courses continue.^13^ In our country, which has very different geographical climates and rich vegetation, the recurrence of rhinitis symptoms and the resulting decrease in the quality of life are especially likely to cause people to resort to complementary and alternative treatment.

Nigella sativa is a traditionally used herb that is locally known as black seed. Nigella sativa seeds are traditionally used in the Middle East and Asia for the prevention and treatment of many ailments.^14^ The biological activity of Nigella sativa is based on the thymoquinone contained in its essential oil. In a clinical study, it was shown that in patients with allergic diseases consisting of AR, bronchial asthma, and atopic eczema, treatment with Nigella sativa oil reduces IgE, eosinophil counts, and endogenous cortisol levels in plasma and urine.^15^ In studies examining the antiallergic effects of the black seed, it was reported that dithymoquinone applied in vitro to peritoneal mast cells of mice could be a reliable compound in the prevention and control of bronchial asthma and other allergic conditions.^16^

The main symptoms of AR in humans are sneezing and an itchy and runny nose. Therefore, animal models with similar allergic symptoms are needed to evaluate the effectiveness of antiallergic drugs. In this study, it was observed that typical AR symptoms, such as sneezing and nose scratching movement, emerged in rats with repeated topical intranasal OVA-protease applications.

In a study conducted by Alsamarai et al., 188 patients (6–45 years of age) with different severities of AR symptoms were given systemic 0.6–0.8 g of Nigella sativa oil three times a day for six weeks and showed the antihistamine potential of Nigella sativa oil.^17^ At the end of six weeks, 96.7% of the group with mild symptoms emerged as symptom-free, whereas 31.8% of the group with moderate symptoms and 22.2% of the group with severe symptoms became symptom-free. In this study, it was observed that the use of Nigella sativa oil for six weeks caused a significant decrease in serum IgE levels. In addition, allergic conjunctivitis, which is generally associated with AR, was found in 66.6% of the patients included in the study, while this rate decreased to 17.6% after six weeks of treatment Nikakhlagh et al. used 0.5 mL of Nigella sativa oil was for four weeks in their study; and no significant difference was found in total serum IgE levels in the study group, but a significant reduction in symptoms was observed in patients with AR symptoms (e.g., itching, nasal congestion, sneezing, and nasal discharge).^18^ Another group of researchers investigated the anti-inflammatory properties of Nigella sativa oil in mice and observed that oral doses of 100, 200, and 400 μL/kg of Nigella sativa oil did not have a significant anti-inflammatory effect, but intraperitoneal injection of the same dose of Nigella sativa oil reduced edema.^19^ In another study by Al-Ghamdi, it was observed that when Nigella sativa oil was given at a dose of 500 mg/kg, it had an anti-inflammatory effect comparable with aspirin at a dose of 100 mg/kg. This study also showed that the analgesic effect of Nigella sativa oil was comparable to aspirin.^20^

The therapeutic effects of N. sativa on allergic diseases are still unclear. An experimental study in mice demonstrated that N. sativa oil caused any changes in Th1 and Th2 cytokine production of splenic mononuclear cells.^21^ In contrast, some studies have shown that it affects Th2 cytokine production in the lung.^22^, ^23^ Gocer et al.^24^ showed that N. sativa oil blocks arachidonic acid metabolism by affecting the 5-lipoxygenase and Cyclooxygenase (COX) pathways and decreases the synthesis of leukotrienes and thromboxane, which are some mediators of asthma and allergies.

Isık et al.^25^ have shown that N. sativa addition to specific immunotherapy significantly improved the symptoms of allergic rhinitis in humans with house dust mites allergic rhinitis according to symptom scores, and immune hematological parameters as lymphocyte subsets and Polymorphonuclear leukocyte (PMN) functions.

In the present experimental study, when we compared the Nigella sativa oil group and the AR groups in terms of the frequency of nose scratching and sneezing, we found that the frequency of nose scratching was significantly lower in the Nigella sativa oil group. We could not detect typical inflammatory changes seen in AR in the Nigella sativa oil group, nor did we detect inflammation in 85.7% of the control group and Nigella Sativa oil group and 71.4% of the mometasone furoate group. We found that inflammation in Nigella sativa oil was found significantly lower compared to the control group. In addition, we found that eosinophil infiltration, cilia loss, chondrocyte hypertrophy, vascular proliferation, and goblet cell increase were significantly decreased in the mometasone furoate and Nigella sativa oil groups compared to the AR group. We observed that the number of sneezing and nose scratching movements decreased significantly depending on the time at which the groups were given mometasone furoate and Nigella sativa oil treatment in accordance with the results in the literature.

## Conclusion

The preventive and healing effects of N. sativa were demonstrated in previously performed animal models of different experimental allergic and respiratory diseases. In this study, the antiallergic and anti-inflammatory efficacy of Nigella sativa oil were investigated in an experimental AR model induced by ovalbumin-protease in rats. We observed significantly similar findings in terms of antiallergic and anti-inflammatory efficacy in the comparison of mometasone furoate and Nigella sativa oil. We suggest that Nigella sativa oil may be used in the symptomatic treatment of AR as well as other medical treatment agents with proven efficacy, however, there is a need for larger-scale studies on this subject.

## Ethical approval

All procedures performed in studies involving animals were in accordance with the ethical standards of Committee in Bagcilar Training and Research Hospital with the approval number: 2018-45 (see Figure, Supplemental Ethical approval photo).

## Data availability

The data that support the findings of this study are available on request from the corresponding author and publisher.

## Authors contributions

RG and MFO conceived and designed research. RG, FE and AT conducted experiments. HDT contributed new reagents or analytical tools. HDT and MG analyzed data. RG and HDT wrote the manuscript. ABC made final controls and english editing. All authors read and approved the manuscript. The authors declare that all data were generated in-house and that no paper mill was used.

## Funding

This research received no specific grant from any funding agency in the public, commercial, or not-for-profit sectors.

## Conflicts of interest

The authors declare no conflicts of interest.
