# Supplementary figures and images for: Effects of nigella sativa oil on allergic rhinitis: an experimental animal study
Source: Braz J Otorhinolaryngol. 2022 Sep 21;88(Suppl 5):S148–55. doi: 10.1016/j.bjorl.2022.09.003 (PMC9801018; doi:10.1016/j.bjorl.2022.09.003)

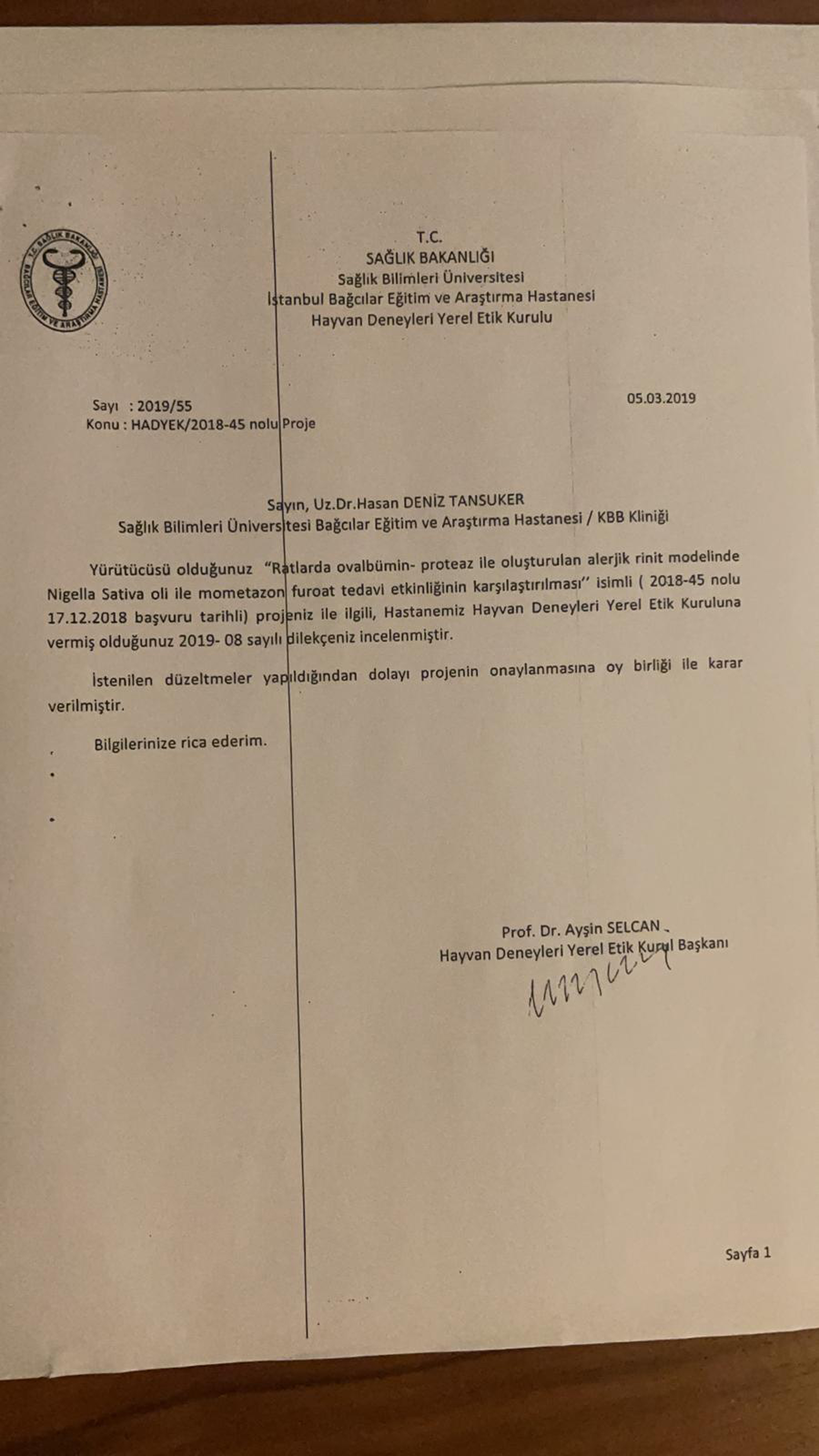

Supplement: Supplementary file 1 [file mmc1.jpg]
